# Health technology assessment in sub-Saharan Africa: a descriptive analysis and narrative synthesis

**DOI:** 10.1186/s12962-021-00293-5

**Published:** 2021-07-07

**Authors:** Samantha Hollingworth, Ama Pokuaa Fenny, Su-Yeon Yu, Francis Ruiz, Kalipso Chalkidou

**Affiliations:** 1 School of Pharmacy, University of Queensland, 20 Cornwall St, Woolloongabba, Brisbane, QLD 4102 Australia; 2 Faculty of Pharmacy and Pharmaceutical Sciences, Kwame Nkrumah University of Science and Technology, Kumasi, Ghana; 3 Institute of Statistical, Social and Economics Research, University of Ghana, Accra, Ghana; 4 National Evidence-Based Healthcare Collaborating Agency, Seoul, Korea; 5 iDSI, London School of Hygiene and Tropical Medicine, London, UK; 6 The Global Fund To Fight AIDS, Tuberculosis and Malaria, Geneva, Switzerland

**Keywords:** Health technology assessment, Sub Saharan Africa, Decision making, Capacity building, Policy making, Priority setting, Narrative synthesis

## Abstract

**Background:**

Countries in Sub-Saharan Africa (SSA) are moving towards universal health coverage. The process of Health Technology Assessment (HTA) can support decisions relating to benefit package design and service coverage. HTA involves institutional cooperation with agreed methods and procedural standards. We systematically reviewed the literature on policies and capacity building to support HTA institutionalisation in SSA.

**Methods:**

We systematically reviewed the literature by searching major databases (PubMed, Embase, etc.) until June 2019 using terms considering three aspects: HTA; health policy, decision making; and SSA. We quantitatively extracted and descriptively analysed content and conducted a narrative synthesis eliciting themes from the selected literature, which varied in study type and apporach.

**Results:**

Half of the 49 papers identified were primary research studies and mostly qualitative. Five countries were represented in six of ten studies; South Africa, Ghana, Uganda, Cameroon, and Ethiopia. Half of first authors were from SSA. Most informants were policy makers. Five themes emerged: (1) use of HTA; (2) decision-making in HTA; (3) values and criteria for setting priority areas in HTA; (4) involving stakeholders in HTA; and (5) specific examples of progress in HTA in SSA. The first one was the main theme where there was little use of evidence and research in making policy. The awareness of HTA and economic evaluation was low, with inadequate expertise and a lack of local data and tools.

**Conclusions:**

Despite growing interest in HTA in SSA countries, awareness remains low and HTA-related activities are uncoordinated and often disconnected from policy. Further training and skills development are needed, firmly linked to a strategy focusing on strengthening within-country partnerships, particularly among researchers and policy makers. The international community has an important role here by supporting policy- relevant technical assistance, highlighting that sustainable financing demands evidence-based processes for effective resource allocation, and catalysing knowledge-sharing opportunities among countries facing similar challenges.

**Supplementary Information:**

The online version contains supplementary material available at 10.1186/s12962-021-00293-5.

## Background

As countries’ political leadership commits to universal health coverage (UHC) in the midst of the well-described demographic and epidemiological transitions and the less well-studied transition from aid, [1] financial pressures are mounting making sustainable financing strategies of the essence for the vision of UHC to become a reality. Whilst economic growth emerges as the main generator of fiscal space for health [2] public resources are still inadequate to cover even the most basic of service packages across sub-Saharan Africa’s (SSA) poorest nations [3]. As a result, choices about priorities must be made; choices that often carry difficult trade-offs. Priority setting—the task of determining the priority to be assigned to a service or individual patient at a given point in time—is unavoidable, since claims (whether needs or demands) on healthcare resources are always greater than the resources available.

Health technology assessment (HTA) is a tool used globally to support explicit, evidence-informed priority setting, and it involves the systematic evaluation of the properties and effects of a health technology, where a health technology can include any intervention that may be used to promote health, to prevent, diagnose, or treat acute or chronic disease, or for rehabilitation [4].

HTA is endorsed by the WHO (WHO assembly HTA in 2014), [5] to inform priority-setting decisions in the context of UHC [4] using context-sensitive evidence to make trade-offs explicit with a consultative process to allow for deliberation and engagement in the decision-making process. HTA has been adopted as a means of priority setting across high income countries (e.g. UK, Australia and Canada) and upper-middle income countries (e.g. Thailand, Brazil and Mexico), whilst other emerging economies such has India and China are establishing their own national institutions. At the same time, high income countries have come together to build HTA coalitions linked to product procurement such as BeNeLuxA (Austria, Belgium, Ireland, Luxembourg, and The Netherlands; beneluxa.org). The International Decision Support Initiative (iDSI) is a global network of health, policy and economic expertise, which seeks to support countries make better decisions about efficient spending on healthcare. iDSI has been working with local partners in SSA since 2013 to promote local capacity and help implement robust HTA processes [6, 7]. There is no single approach to building HTA institutions. It requires policies setting out preferred methods and processes, stakeholders, timelines, key audiences, and expectations around how HTA evidence will or should be used in routine decisions [8].

In this context, we want to better understand how priority setting decisions for spending in healthcare are made in SSA, with specific reference to the use of HTA. Our aim in this study was to undertake a systematic review of the literature pertaining to policies and capacity building in relation to HTA in sub-Saharan Africa.

## Methods

We systemically searched the medical literature in Pubmed, EMBASE, Scopus, EBSCO, and ABI/INFORM Global, and hand searching up to 26 June 2019. We used terms including Sub Saharan Africa, health technology assessment (HTA), economic evaluation, capacity building, and policy. We used the thesaurus for each database: Medical Subject Headings (MeSH) for Medline; and EMTREE for Embase (Table 1).

**Table 1:** Search terms

Topic	Level	Terms
HTA	1	"Technology Assessment, Biomedical"[Mesh] OR HTA[Title] OR technology assessment*[Title]
	2	Technology assessment*[Title/Abstract] OR health technolog*[Title/Abstract] OR knowledge synthes*[Title/Abstract] OR research eviden*[Title/Abstract] OR ((evidence-based[Title/Abstract] OR evidencebased[Title/Abstract] OR evidence-informed[Title/abstract]) AND (guide-line*[Title/Abstract] OR guideline*[Title/Abstract]))
Health policy, decision making	1	"Policy Making"[Majr] OR "Health Policy"[Majr] OR "Decision Making"[Majr] OR "Health Priorities"[Mesh] OR "Health Plan Implementation"[Mesh] OR "Health Services Administration"[Mesh] OR decision-mak*[Title] OR policy decision*[Title]
	2	Decision-mak*[Title/Abstract] OR health care decision*[Title/Abstract] OR health care polic*[Title/Abstract] OR health polic*[Title/Abstract] OR health practic*[Title/Abstract] OR clinical decision*[Title/Abstract] OR clinical intervent*[Title/Abstract] OR clinical practic*[Title/Abstract] OR professional practic*[Title/Abstract] OR policy mak*[Title/Abstract] OR policy decision*[Title/Abstract] OR policy question*[Title/Abstract] OR reimbursement decision*[Title/Abstract]
SSA	1	Exp Africa (*NB includes non-SSA countries*)
	2	Africa Angola Benin Botswana “Burkina Faso” Burundi Cameroon “Cape Verde” “Central African Republic” Chad Comoros Congo “Cote d’Ivoire” Djibouti “Equatorial Guinea” Eritrea Ethiopia Gabon Gambia Ghana Guinea Guinea-Bissau Kenya Lesotho Liberia Madagascar Malawi Mali Mauritania Mauritius Mozambique Namibia Niger Nigeria Reunion Rwanda “Sao Tome and Principe” Senegal Seychelles “Sierra Leone” Somalia “South Africa” Sudan Swaziland Tanzania Togo Uganda “Western Sahara” Zambia Zimbabwe)

**Fig. 1:**
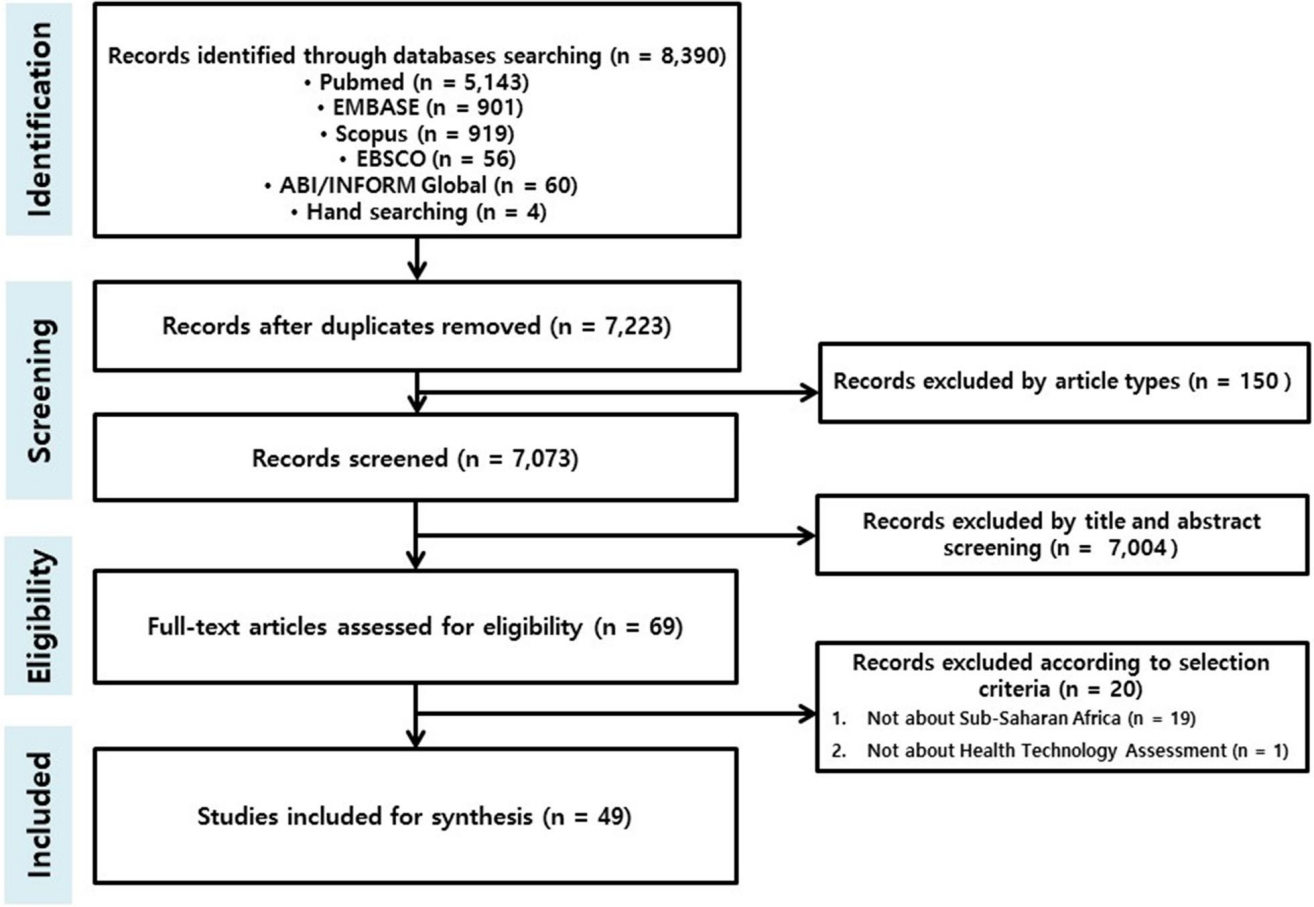
Flow diagram of articles extracted in the review

Papers were eligible for inclusion if they reported on HTA or approaches that considered priority-setting, decision making process or healthcare policy reform. Eligible papers also need to report at least on low-income or lower middle-income country (LMIC) as defined by the World Bank [9]. We compiled the peer-reviewed papers or conference abstracts which were published in English. We specifically excluded published economic evaluations of interventions in the SSA context. Two researchers independently conducted the study selection. First, the reviewers screened the relevance of the articles by title and abstract. Some articles were excluded by study type such as theses, serials, and newspaper articles. Then, we obtained the full text of the articles and judged the eligibility of the inclusion. Any differences were resolved by consensus with input from other authors. The selection process was performed in accordance with the Preferred Reporting Items for Systematic Reviews and Meta-Analyses (PRISMA) statement (Fig. 1) [10]. Relevant data were extracted into an Excel file and, together with the selected references, were stored in a secure shared folder that all authors could access.

We quantitatively extracted and descriptively analysed information from the selected publications: country, authors and affiliations, type of study and methodological approach, type of technology, and study participants. We used a parallel-results convergent synthesis design, which involves a process where the synthesis of both quantitative and qualitative elements in the review process is concurrently conducted; both the quantitative and qualitative papers are given equal importance and the results are interpreted together [11]. Given the evidence base consisted of multidisciplinary approaches and various study types there were challenges in applying quality criteria. Hence the assessment of each study focussed on relevance to the topic rather than quality of the study [12].

## Results

### Descriptive analysis

#### Study types

We included 49 papers in the review (Table 2 and Additional file 1: Table S1); 26 (53%) were primary research, 17 (35%) were reviews, and there were six ‘others’ (12%, editorial, letter, forum, perspective). Of the primary research there were 15 qualitative studies (31%, using interviews), four quantitative studies (8%, three using surveys) and seven papers (14%) used mixed methods—four used surveys and other methods and three were discrete choice experiments. Of the 17 reviews, there were 12 narrative reviews (24%) and five systematic reviews (10%).

**Table 2:** Articles by first and corresponding authors: country groupings and institutions

	Academic		Government		Other		Not stated		TOTAL (N = 49)	
	n	%	n	%	n	%	n	%	n	%
First author										
SSA	20	41	4	8%					24	49
America	9	18			2	4%			11	22
Europe/UK	8	16	1	2%	1	2%			10	20
Other	2	4					2	4%	4	8
Total	39	80	4	8%	3	6%	2	4%	49	100
Corres. author										
SSA	14	29	3	6%	1	2%			18	37
America	5	10			3	6%			8	16
Europe/UK	15	31	2	4%	3	6%			20	41
Other	1	2					2	4%	3	6
Total	35	71	5	10%	7	14%	2	4%	49	100

SSA: Sub-Saharan Africa; UK: United Kingdom

#### Countries

Most papers only referenced a single country (n = 27, 55%). Two papers each referred to two, three and 12 countries and one paper each referred to four, seven, nine, and ten countries. Twelve papers (24%) did not provide specific information on a particular county, for example general information on SSA or LMIC, review, conference abstract, etc. Of the 34 papers (69%) referring to specific SSA countries, South African was referred to the most (n = 17), followed by Ghana (n = 8). Five countries represented more than half (58%) of all the papers: South Africa, Ghana, Uganda (n = 5), Cameroon (n = 4), and Ethiopia (n = 4). There were two papers each from Democratic Republic of Congo, Kenya, Nigeria, and Rwanda. There was one paper each from Benin, Botswana, Burkina Faso, Chad, Cote d’Ivoire, Gabon, Guinea, Namibia, Niger, Senegal, Swaziland, Zambia, and Zimbabwe.

#### First and corresponding or last authors

Half of the first authors (n = 24, 49%) were from SSA countries (Table 2). Of the first authors, most were academics from universities or research institutes (n = 20) followed by government employees (n = 4). Most of the non-SSA authors were from US (n = 11) and Europe or UK (n = 10). More than one in three corresponding authors (n = 18, 37%) were from SSA countries; many were from Europe and UK (n = 20, 41%). Of the SSA authors, most (n = 14) were academics. The corresponding authors were distributed across more countries than first authors.

#### Participants and technology

We examined the participants in the empirical papers; among the 26 studies which conducted interviews or surveys, most informants were policy makers (n = 21). The remaining five were either health professionals or not specified in detail (e.g. global stakeholders, key opinion leaders). Most studies (n = 35, 71%) were not related to any specific technology. One in four papers (n = 12, 24%) concerned pharmaceuticals and two papers (4%) concerned medical devices.

### Narrative synthesis

Five main themes emerged from the literature relating to HTA in SSA settings: (1) the use of HTA; (2) decision-making in HTA; (3) values and criteria for priority areas in HTA; (4) involving stakeholders in HTA; and (5) progress in HTA in SSA.

1. Use of HTA in SSA
  SSA is largely characterised by (LMICs) with limited healthcare budgets struggling to prioritise healthcare needs and direct their investments in health technologies. The main challenges to HTA in SSA are: i) little use of evidence in policy-making; ii) low awareness due to weak or non-existent local institutions with the capacity to conduct HTA; iii) limited technical expertise; and iv) a lack of local data and appropriate tools to conduct HTA [13, 14]. Countries have, however, articulated how HTA would be used in policy. For example, Babigumira et al. (2016) [14] found that Rwanda and South Africa sought to use HTA for a range of regulatory, coverage or formulary decisions, and reimbursement activities. In contrast, Ethiopia and Namibia reported that HTA would not be used for reimbursement purposes. Nonetheless, there is a growing concern about the use of HTA in SSA with varying levels of institutional development and limited application to making regulatory, coverage or formulary and reimbursement decisions [14, 15].
  Kachieng'a and Boonzaier (1999) noted that HTA was a key means to assess technologies and manage efficiency, highlighting the unique ability of HTA to change provider behaviour based on evidence. However, they cautioned against using HTA to resolve equity issues and disparities in health outcomes within the South African health system [16]. Similarly, a qualitative study in Botswana reiterated the imperative for systems thinking to promote access to medicines, efficient delivery, and cost-effectiveness when implementing health financing reforms to make progress towards universal health coverage [17].
  (i) Little use of evidence and research in making policy
    Research findings are increasingly becoming an important input in the health policy process. Arguably most health researchers and those who fund health research would like to see these activities influencing actual policy decisions, the goal being improvements in health care delivery based on the evidence. In most SSA countries, however, there are growing concerns that research findings are not adequately utilised by health policymakers. In Tanzania, for example, decisions about the selection of essential medicines were made by committees of experts who largely used experience and discretionary judgement, with evidence playing only a limited role in decision-making process [18]. In this context the translation of research evidence into drug policy was often constrained by poor communication between researchers and policy makers; individual perceptions or attitudes towards the drug, and the reluctance by some decision-makers to approve changes based on anticipated resistance to implementation [19]. In addition, reforms to pharmaceutical regulation in Tanzania were thwarted by vested interests [20].
    The factors influencing the use of research findings by health policy-makers in Mali were access to information, relevance of the research, duration of the research, trust in the research methods and outputs, and the level of authority of researchers [21].
    Adopting a conceptual framework and tool that takes into account individual and organisational constructs, Rodriguez et al. (2017) assessed the capacity within ministries of health (MoHs) to demand and use research evidence for decision-making in eight LMICs (two countries in SSA) [22]. Both South Africa and Zambia had the highest level of organisational capacity to use research; individual capacity was seen as high in South Africa [22]. Such capacity can be supported by targeted training interventions. In Nigeria, for example, training workshops of evidence-informed policymaking, which included both researchers and policymakers, improved policymakers' knowledge and competence to use evidence in the policy process [23].
    A review of the state of health economic and pharmacoeconomic evaluation research in Zimbabwe concluded that the use of such research “was low, and 31% of the studies were of poor quality. More and better quality health economic research in Zimbabwe is warranted.” [24]. In this situation policy makers would be less inclined to use local studies and perhaps rely more on international evidence, if evidence is considered at all. Reviewing progress towards UHC in francophone Sub-Saharan Africa, Paul et al. (2018) assessed global health experts’ confidence in policy options. The limited use of HTA stems from a lack of consistent evidence that discourages use in policymaking. In the ideal scenario, they argue that experts' opinions should be framed within strengthened inclusive and "evidence-informed deliberative processes" where the trade-offs along the three dimensions of UHC (extending the population covered against health hazards, expanding the range of services and benefits covered, and reducing out-of-pocket expenditures), can be discussed in a transparent and contextualized setting” [25].
  (ii) Low awareness of HTA and economic evaluation
    There is limited understanding of the basic concepts of HTA or of the relevance of economic evaluation to decision making in the health sector. With many countries making UHC a priority, there is a window of opportunity for greater use of HTA in policy and decision-making in SSA [26]. The lack of awareness and understanding of the principles of HTA and its impact on the improvement of health care are among the many challenges faced by decision makers and stakeholders. HTA can very much depend on the economic evaluation of alternative health interventions in a given context but inadequate attention to this aspect restricts the use of HTA as evidence for decision-making. Low HTA awareness and a decision making culture that relies on historical allocations or traditional hierarchies of power and policy making, are recognised as critical bottlenecks for poor economic evaluation practice in the Ethiopian health sector [27]. This perhaps explains why HTA is more prevalent among developed countries (e.g. U.K, Europe, Australia) where it used extensively to inform healthcare policy decisions, enhance program management, and assess new technologies [27]. In SSA, however, there is a growing trend where HTAs are systematically used to evaluate formulary inclusion and as a method of cost containment [28]. Countries in SSA can act as change agents to raise awareness and knowledge of the benefits of using HTA in other countries in the sub-region [26, 29].
  (iii) Low levels of skills, expertise and training in HTA
    Healthcare systems are dynamic and never complete [30] so acquiring relevant skills is vital in adapting to change. HTA can offer a way to address inefficiencies in health systems and create a broad understanding of the impact of technologies on policy. Mueller et al. have argued that the key to reduce the skills gap is training through regular courses and conferences. The lack of trained people and relatively low levels of understanding of HTA processes and their usefulness are major barriers to HTA uptake [15]. A survey of participants in a workshop comprising HTA agencies, networks, and training organisations from across the world concluded that even though various HTA training programs existed in industrialised countries (either institutionalised or demand-based), they were virtually non-existent in African countries [29].
    The structure of HTA programs varies across jurisdictions according to decision-maker needs. Effective decision-making should include multiple criteria (medical, economic, technical, ethical, social, legal, and cultural) and requires multi-disciplinary teams of experts to produce assessments [29]. These disparate HTA domains and the transdisciplinary approach infuses this policy research area with a complexity that requires professionals with certain skills and competencies. Three broad profiles were considered critical to establish an HTA initiative: clinical scientists, economists, and information scientists [29]. Beyond this, there are ongoing debates as to whether to professionalise HTA so that certain skills could differentiate an HTA professional. In view of this, further scientific investigation would be needed including active engagement of stakeholders to activities such as listing and defining basic core competencies and secondary competencies [29].
  (iv) Lack of local data and appropriate HTA tools
    HTA demands integrating information from multiple sources in order to assess value, however defined. Locally-relevant data across several dimensions are needed to analyse even relatively simple technologies but these data may not be available [31]. Many parameters of decision-making are sociopolitical in nature and sometimes difficult to quantify (e.g. decisions about spending on primary vs tertiary health care or the relative merits of preventive, curative, or rehabilitative care). The reprioritisation of health services and the process of structural change need to be carefully and comprehensively planned [32]. Evaluations of efficiency and cost effectiveness in a limited-resource context such as Cameroon should be a priority agenda for all stakeholders including high-level health sector decision-makers, program managers, and users [33]. Not all technologies warrant a full assessment, nor is it feasible to provide comprehensive assessments for all technologies.
    Most SSA countries with the greatest need for HTA have a limited informational basis that is context relevant for making evidence-based choices. Earlier surveys in South Africa highlighted data issues that are also relevant for most SSA countries such as the absence of a systematic collection of data to determine the actual cost of a technology in the healthcare system [31]. The accounting systems of most hospitals do not allow for the calculation of the cost of an individual technology or medical device. This information is vital to determine the real costs and hence cost effectiveness of the technology. For example, there is limited information on the cost to hospitals of using inappropriate health care technology or failing to properly maintain equipment, even though the WHO asserts that these factors contribute to the high cost of technology in developing countries [31]. Selected agencies are, however, considering NICE decisions as a factor for rejecting or restricting the use of drugs, which in other cases would be recommended or reimbursed [34].
2. Decision-making and HTA
  Generally, health system stewards in both the public and private sectors are expected to apply the tools of regulation, governance, and accountability towards maximizing population health in an efficient and cost-effective manner [17]. As third-party payment (insurance) systems develop, payers can use HTA to ensure that claims are justified, focusing on the highest cost interventions and using cost-effectiveness analyses to manage demand for services [14]. When healthcare plans are large or there are national health systems, decision-making becomes more centralised with greater emphasis on efficiency. All healthcare technologies, including broader societal objectives for the system, become candidates for HTA.
  While HTA guidelines may exist in some SSA countries, there were no examples of a formal, dedicated, and independent HTA institution in SSA [14, 35, 36]. Previous health technology management (HTM) policies have not incorporated the views of low-level actors such as equipment users and technicians, even though they face most of the problems concerning HTM decision making [37]. Regulation of medical devices in developing countries is not as established as found in developed countries and so a higher degree of variability is common [38]. The authors suggest a comprehensive and integrated regulatory framework approach using the South African health technology policy framework to compare and benchmark devices and so improve the safety and efficiency of medical devices [38].
  In South Africa, the criteria for selecting essential medicines for the Essential Medicines List (EML) showed the process has been refined over the years as revealed by in-depth interviews with members of the national EML committee.
  It is now predominantly an evidence-based process where the first considerations are the quality, safety, and efficacy of a medicine followed by cost considerations comprising economic evaluations and pricing [39]. Although Ghana has made progress in formalising HTA for decision making in the health system [36, 40] as countries within the sub-region move towards formal adoption of HTA, we will need to assess the knowledge and attitude of potential users and producers of HTA. In the context of UHC there is growing need to institutionalise decision-making and priority setting with an example of devising the government’s agenda for National Health Insurance in South Africa [41].
  Decisions to adopt new vaccines are, by nature, political as revealed in a qualitative study of national decision-making processes in seven LMICs (including five SSA countries) on new vaccine adoption. Despite this, it is clearly important that evidence is used to inform these decisions and that the feasibility and sustainability of introducing new vaccines are considered [42].
  HTA necessarily requires an estimate of the ‘value for money’ of an intervention, and this is usually informed by the use of a cost-effectiveness threshold as part of the decision-making process [43]. Such an exercise is fraught in LMIC settings, and three approaches encompass the following: affordability, a function of gross national income, and eliciting preferences. There is no clear guidance on this topic but the valuation approach needs to be appropriate for the setting [43].
3. Values and criteria for priority areas in HTA
  There is an apparent absence of HTA tools to systematically assess health technologies relevant to the SSA context but two tools (Know essentials and multi-criteria decision analysis (MCDA)) could be combined for use [44]. An appropriate HTA tool for resource-constrained settings should address all the important criteria of decision making in a given context. It must also recognise technical and other constraints that exist when implementing HTA processes in a particular setting. Tool development, especially in relation to setting out important decision criteria, relies on local engagement to define a context-specific methods framework, such as a Reference Case for economic evaluation [45]. In comparing decision-makers’ preferences at the country level, group preferences of policymakers show explicit but varying trade-offs with respect to efficiency versus equity, reflecting their diverse settings [46]. A successfully implemented HTA approach would be transparent about such trade-offs.
  In Ghana, a pilot study using MCDA to rank different interventions showed the potential feasibility of accounting for efficiency, equity, and other societal concerns in prioritising decisions [47]. There is anecdotal evidence that health policymakers in Ghana used evidence in the process of developing policy for the health sector’s third Programme of work [48]. Agreeing on common criteria for priority setting among key stakeholders in Uganda can be a challenge; they may have differing opinions on which criteria to adopt [49]. There was an overlap in the elements of fairness and accountability as key criteria for priority selection across health care systems and levels of decision making [50]. In the context of austerity policies in Cameroon, rationing became one criterion in selecting treatments for hepatitis C, showing how different values become applicable in different settings [33]. Overall, priority-setting at the country level requires a commitment to improve equitable access to, and appropriate use of, medicines by using information available in the system (e.g. price, availability, quality, utilisation, registration, and procurement) in a transparent way; engaging both public and private stakeholders [51].
  Nearly all the health care coverage schemes in a study of decision-making in 25 LMICs (Ghana and Uganda in SSA) involved various representatives and stakeholders in their decision-making processes [52]. Decisions were based on disease areas (e.g. maternal and child health and non-communicable diseases) using evidence from the literature and the coverage policies of other schemes but funding was the most commonly reported reason for restricting coverage [52].
4. Involving stakeholders in HTA
  The major HTA stakeholders are public sector policymakers with substantial influence over health system regulation and resource allocation. They could also include those in a position to take key decisions beyond the health sector [30]. Other stakeholders include health professionals, academics, and members of the community. The selection of stakeholders for an HTA topic will depend on the purpose of the assessment and available resources, among other factors. A comprehensive HTA process will engage with range of public and private stakeholders, but in many cases numbers will be constrained by the costs of undertaking such levels of engagement. In Uganda, key stakeholders who might be ultimately involved in CEA (but have had little exposure to it) included health care workers, technical officers at the Ministry of Health (directly involved in policy formulation), post-graduate students training in health-related fields, and academics in university medical (and other) schools [53].
  Best practice suggests a need to involve the wider community in their role as health service users, but their participation in decision-making bodies to date is generally very low. National health planners and expatriate consultants do not always recognise the need for a detailed analysis of the relationship between community participation in health and the quality and quantity of health care [54]. One study from Uganda explored the use of burden of disease and cost-effectiveness in district health planning and the effect on budgets and actual expenditure [55]. Actual budget allocations seemed to diverge from such evidence. From the study, other context-specific, non-technical considerations were also part of the process, so the authors suggested that the "technical" issue of efficiency needed to be better understood and integrated in the concept of an accountable health care system at the district level. This could be accomplished by increasing the involvement of the peripheral parts of the health care system; most likely the target population [55].
  HTA in SSA settings must engage with a complex array of stakeholders, network with other research organisations, build partnerships with different levels of government and train the future generation of HTA researchers and policy-makers [35]. High-income countries, LMICs, and donors have all recognised that the views and values of health service end users should be included in policy formulation, planning and implementation [53]. Different stakeholders including national governments, global donors, the commercial sector, and service delivery institutions must work together to address the growing burden of cancer across economies of low, middle, and high income [56]. Engagement among countries of the Global South can provide a supportive platform to share knowledge that is more applicable and pragmatic [13].
5. Progress in HTA in SSA
  Despite the challenges faced by SSA countries in implementing HTA, there are a few success stories. Some countries have used HTA-derived information in decision-making and developed related guidelines. However, HTA development is characterised by fragmentation, and often takes place in the absence of an institutionalised framework involving a formal dedicated body or unit. For example, although the National Department of Health in South Africa has published HTA frameworks and strategies since the 1990s, there is no formalised system for routinely using HTA in decision making to improve health outcomes and health service delivery [26]. The National Immunisation Technical Advisory Group in South Africa decides on the introduction of new vaccines. They consider WHO guidance and recommendations but the decision to introduce a new vaccine is based on local data [57]. In Ethiopia, the Health Economics and Financing Analysis (HEFA) team was established to meet the increasing the need for HTA within the Ministry of Health [58]. HEFA is tasked with leading the application of evidence-based healthcare decision-making by organising available evidence, costing interventions, defining effectiveness measures of the different health programs, and supporting policy makers at the national and regional levels [58].
  Transparency of HTA processes is integral to good decisions. One study aimed to quantitatively benchmark HTA agencies in ten countries (mostly HIC but also included South Africa) for attributes of transparency [59]. Drummond and co-authors concluded that transparency is a key attribute in driving HTA maturity [59]. The more established the HTA agency, the higher the transparency in the submission process, pricing policies, pharmacoeconomic requirements, and approach.

## Discussion

We identified 49 papers reporting on HTA in sub-Saharan Africa. Half of the papers were primary research studies and they were mostly qualitative. A third of the papers were reviews, mostly narrative reviews including five systematic reviews. Most papers only referenced a single country while around 25% did not provide specific information relating to a particular country. Five countries represented nearly six in ten studies: South Africa, Ghana, Uganda, Cameroun and Ethiopia. Half of the first authors were from SSA countries and most were from universities or research institutes. Half of the studies used surveys or interviews and most informants were policy makers. The narrative review identified five main themes. The first theme concerned the use of HTA in SSA, highlighting the low levels of awareness and uptake. The other four themes considered decision making in HTA, values and criteria for setting priorities for HTA, HTA stakeholders, and examples of progress in HTA in SSA.

This is, to our knowledge, the first comprehensive analysis of the published literature relating to HTA in SSA incorporating both quantitative and qualitative analyses. The main limitation of the study is that it only captured papers published in the academic literature in English and did not include those documents in the grey literature (reports, technical documents, etc.) or otherwise not publicly available. We surmise that this literature could be quite extensive.

This study extends previous work exploring the health landscape [60] and economic evaluations [61] in SSA and some country-specific articles [7]. Certainly, countries in SSA can take note of progress regarding HTA in other LMICs [8, 62–65]. A recent study examined the knowledge and attitude of Ghanaian decision-makers and researchers towards HTA (interviewed in 2016 but only recently published so not included in our review) [66]. Participations accepted the usefulness of HTA to develop treatment guidelines, inform resource allocation, contain costs, and ensure value for money. The authors pointed to the lack of suitable data to inform HTA in Ghana; current data were inadequate, fragmented, inaccessible, and not available in a usable form for any HTA exercise. A more recent article explored the views of Nigerian stakeholders about HTA; use was limited due to low capacity to produce and use HTA [67]. Efforts to institutionalise HTA in South Africa noted the importance of political support, local capacity and awareness of HTA [68].

The significant obstacles of limited skills capacity, political will, and governance, plus data gaps stymie countries’ attempts to create legitimate evidence-informed priority setting institutions. There is a strong trend, however, to change the ‘business as usual’ approach to decisions about allocating resources with four contributory factors.

Firstly, global agencies are actively supporting HTA on the road to UHC as evidenced by resolutions from the WHO [5] and the United Nations General Assembly [69]. The Global Fund to Fight AIDS, Tuberculosis and Malaria (GFATM) plans to work with established HTA agencies such as NICE in the UK and HITAP in Thailand, and possibly with other organisations such as the GAVI Alliance (formerly the Global Alliance for Vaccines and Immunisation), on product selection and cost-effectiveness analyses [70, 71]. Our systematic review reveals a growing literature from across SSA with countries such as South Africa [68, 72] and Ghana [73, 74] legislating to institutionalise HTA.

Secondly, aid transition may offer an opportunity to support this trend to make evidence-informed and fair trade-offs, given limited resources for UHC. This can be accomplished through institutional partnerships across North–South and South-South; support for useful data collection [75, 76]; capacity building [8]; and alignment by development partners such as GFATM [77] to these same principles of evidence-informed priority setting. Donors are urged to support institutional strengthening in light of aid transition [78, 79].

Thirdly, countries are building coalitions to take advantage of economies of scale and scope, perhaps aligned to efforts of the African Union Development Agency for regulatory harmonisation [80]. A particularly challenging area is the procurement of health commodities where countries may have previously benefited from donor-aggregated demand and pooling mechanisms, negotiated prices, purchasing, and delivery. Extending the regulatory harmonisation to procurement and incorporating HTA to provide evidence of comparative clinical and cost effectiveness [81] would be part of a broader and needed strategy to improve health care and, by extension, health outcomes in SSA. This especially important as countries seek to achieve UHC and as they navigate a very challenging post-Covid-19 fiscal context [82, 83].

Fourthly, HTA provides a structured way, involving deliberative processes, [84, 85] to bring together evidence of both clinical and cost effectiveness to inform priority-setting activities.There is a need to involve many stakeholders including (government) decision makers, clinicians, academics, consumers, development partners, and HTA knowledge brokers [35]. In particular, collaborations between local researchers and policy makers will be critical in building capacity to produce and interpret policy relevant research. Effective partnerships between local and international researchers plus key government stakeholders can leverage existing skills and knowledge to generate a critical mass of individuals and institutions [35]. Global development partners also have an important role in supporting HTA institutionalisation, especially given the apparent differential access to—and use of—evidence between donor-supported programs relative to less high profile programs within a country [86]. This implies a comprehensive view of what constitutes appropriate capacity building [8]. For example, while an important element, global support has to be more than facilitating the delivery of training courses or participation in international conferences. The International Decision Support Initiative (iDSI) is a global network of health, policy and economic expertise, which seeks to support countries make better decisions about efficient spending on healthcare [87]. iDSI has been working in Africa since 2013 to develop local capacity and support implementation of robust HTA processes. iDSI core partners have supported collaborations between Thailand and Kenya [88], and Norway and Ghana [73, 74] in relation to strengthening value for money decisions (and the capacity to make them) as part of UHC, a key emphasis is on building relationships that focus on people, policy, and process for implementing HTA [7]. HTA methods and processes can be adapted to inform policy decisions while accounting for uncertainty considerations and context-specific practicality constraints in LMIC [89, 90].

This analysis provides a baseline to inform future capacity building for HTA in SSA. We consider it worthwhile to engage HTA proponents in SSA to release their unpublished reports perhaps to a common online space (e.g. hosted by iDSI) to enable and promote South-South driven knowledge exchange [13, 64]. We will review and provide research capacity building [32] in selected SSA countries as integral to ongoing iDSI work. We seek to promote recently-developed tools for priority setting [91] and HTA [92]. Francophone Africa needs to be included in the process [25]. This review has focussed on SSA but subsequent studies could incorporate efforts in North Africa [93–95].

## Conclusions

There has been growing interest in HTA in SSA countries, motivated in part by commitments to UHC and greater co-financing requirements amid a changing donor landscape. However, HTA awareness remains low, and HTA-related activities are uncoordinated and often disconnected from policy. Further training and skills development is needed, but this has to linked to a strategy focusing on strengthening within-country partnerships, particularly among researchers and policy makers, but also including other stakeholders. The international community has an important role here, supporting policy- relevant technical assistance, highlighting that sustainable financing demands evidence-based processes for effective resource allocation, and catalysing knowledge sharing opportunities between countries facing similar challenges.

## Supplementary Information

**Additional file 1: Table S1. **Details of included studies: countries covered, country and institution of first author, country and institution of corresponding author type of technology, publication type study type, research methods and participants

## Data Availability

The datasets used and/or analysed during the current study are available from the corresponding author on reasonable request. The articles selected in this review are available in the published literature.
