# Supplementary material for: Health technology assessment in sub-Saharan Africa: a descriptive analysis and narrative synthesis
Source: Cost Eff Resour Alloc. 2021 Jul 7;19:39. doi: 10.1186/s12962-021-00293-5 (PMC8261797; doi:10.1186/s12962-021-00293-5)
Supplement: Supplementary file 1 — Additional file 1: Table S1. Details of included studies: countries covered, country and institution of first author, country and institution of corresponding author type of technology, publication type study type, research methods and participants [file 12962_2021_293_MOESM1_ESM.docx]

**Table S1. Details of included studies: countries covered, country and institution of first author, country and institution of corresponding author type of technology, publication type study type, research methods and participants**

| **Name** | **Countries** | **Country - first author** | **Institution -first author** | **Country  - corres OR last author** | **Institution - corres OR last author** | **Technology** | **Publication type** | **Study type** | **Research method** | **Participants** |
| --- | --- | --- | --- | --- | --- | --- | --- | --- | --- | --- |
| Achoki et al, 2017 | Botswana | USA  /Netherland | Academic | Netherland | Academic | NA | Primary research | Qualitative | Interview study | Policy maker |
| Albert et al, 2007 | Mali | Norway | Academic | Mali | Government | pharmaceuticals | Primary research | Qualitative | Interview study | policy maker global expert |
| Azefor, 1989 | SSA: Guinea, Cote d'Ivoire, Ghana, Nigeria, Cameroon, Senegal, Niger, Burkina Faso, Mali | USA | World Bank | NA | NA | NA | Review | Narrative review | NA | NA |
| Babigumira et al,  2016 | SSA: Congo, Ethiopia, Jordan, Kenya, Namibia, Rwanda, South Africa, Swaziland | USA | Academic | USA | Academic | NA | Primary research | Mixed | Cross sectional survey | policy maker, academic, NGO |
| Baltussen et al, 2006 | Ghana | Netherland | Academic | Ghana | Consultant | NA | Primary research | Mixed | Discrete choice experiments | policy maker |
| Bigdeli et al, 2013 | SSA: Suriname, Chad, Cameroon, Congo, Gabon, Rwanda, Ghana | Switzerland | WHO | Switzerland | WHO | pharmaceuticals | Primary research | Qualitative | Interview study | global stakeholders (not specified) |
| Bolsewicz Alderman, et al, 2013 | LMIC not specified | Australia | Academic | Australia | Academic | NA | Other | Forum | NA | NA |
| Burchett et al, 2012 | SSA: Cameroon, Ethiopia, Kenya, Mali, South Africa | UK | Academic | UK | Academic | pharmaceuticals | Primary research | Qualitative | Interview study | policy maker, academic, NGO |
| Chabrol et al, 2017 | SSA: Cameroon | France | Academic | France | Academic | pharmaceuticals | Primary research | Qualitative | Interview study | policy maker, health professionals, global expert, NGO, patient |
| Chalkidou et al, 2014 | SSA not specified | UK | Government | UK | Academic | NA | Review | Narrative review | NA | NA |
| Diaconu et al, 2017 | LMIC not specified | UK | Academic | UK | Academic | medical device | Review | Systematic review | NA | NA |
| Doherty, 2010 | South Africa | South Africa | Academic | NA | NA | NA | Review | Narrative review | NA | NA |
| Doherty et al, 2017 | Africa, not specified | South Africa | Academic | South Africa | Academic | NA | Review | Narrative review | NA | NA |
| Doherty & Bloom, 1996 | South Africa | South Africa | Academic | UK | Academic | NA | Review | Narrative review | NA | NA |
| Gavaza et al, 2008 | Zimbabwe | USA | Academic | USA | Academic | pharmaceuticals | Review | Systematic review | NA | NA |
| Gutierrez et al, 2015 | SSA: Ghana | USA | Academic | USA | Academic | NA | Primary research | Quantitative | Cross sectional survey |  |
| Hall et al, 2017 a | Ghana | Australia | No information | Australia | No information | NA | Primary research | Qualitative | Interview study | policy maker, academic |
| Hall et al, 2017 b | Ghana | Australia | No information | Australia | No information | NA | Review | Systematic review | NA | NA |
| Hernández-Villafuerte et al, 2014 | SSA: Ghana | UK | Government | UK | Government | pharmaceuticals | Primary research | Mixed | Survey/Policy review | key opinion leaders (not specified) |
| Hofman et al, 2015 | South Africa | South Africa | Academic | UK/Canada | Academic | NA | Other | Editorial | NA | NA |
| Houngbo et al, 2017 | Benin | Benin | Academic | Netherland | Academic | NA | Primary research | Mixed | Survey/Interview | policy maker, health professional, global expert |
| Jehu-Appiah et al, 2008 | Ghana | Ghana | Government | Ghana | Government | NA | Primary research | Mixed | Discrete choice experiments | policy maker |
| Jeppsson et al, 2004 | Uganda | Sweden | Academic | Sweden | Academic | NA | Primary research | Qualitative | Interview study | policy maker |
| Kachieng'a et al, 1999 | South Africa | South Africa | Academic | South Africa | Academic | NA | Review | Narrative review | NA | NA |
| Kapiriri et al, 2004 | Uganda | Norway | Academic | Norway | Academic | NA | Primary research | Quantitative | Cross sectional survey |  |
| Kapiriri et al, 2009 | SSA: Uganda | Canada | Academic | Canada | Academic | NA | Primary research | Qualitative | Interview study | policy maker |
| Kriza et al, 2014 | SSA not specified | Germany | Academic | Germany | Academic | NA | Review | Systematic review | NA | NA |
| Mazumder et al, 2015 | SSA: South Africa | India | Consultant | USA | Consultant | NA | Primary research | Quantitative | Cross sectional survey |  |
| Miot et al, 2017 | SSA: South Africa | South Africa | Academic | Germany | Scenarium Group GmbH | pharmaceuticals | Other | Perspective | NA | NA |
| Mirelman et al, 2012 | SSA: Uganda | USA | Academic | UK/USA | Academic | NA | Primary research | Mixed | Discrete choice experiments | policy maker |
| Mori et al, 2014 | Tanzania | Tanzania/Norway | Academic | Norway | Academic | pharmaceuticals | Primary research | Qualitative | Interview study | policy maker |
| Mori et al, 2013 | Tanzania | Tanzania/Norway | Academic | Tanzania | Academic | pharmaceuticals | Primary research | Qualitative | Interview study | policy maker, health professionals, NGO |
| Mubyazi et al, 2005 | Tanzania | Tanzania | Government | Switzerland | WHO | pharmaceuticals | Primary research | Qualitative | Interview study | policy maker, academic |
| Mueller et al, 2011 | South Africa | South Africa | Academic | South Africa | Academic | NA | Other | Letter | NA | NA |
| Mueller et al, 2016 | Not specified | South Africa | Academic | Italy | Academic | NA | Review | Narrative review | NA | NA |
| Mueller, 2017 | South Africa | South Africa | Academic | NA | NA | NA | Primary research | Mixed | Survey/Policy review | policy maker, academic |
| Müller et al, 1994 | South Africa | South Africa | Academic | South Africa | Academic | NA | Other | Editorial | NA | NA |
| Musuuza et al, 2014 | Uganda | USA | Academic | Uganda | Academic | NA | Primary research | Quantitative | Cross sectional survey | policy maker, health professionals, PG students |
| Ngcobo et al, 2012 | South Africa | South Africa | Government | South Africa | Academic | pharmaceuticals | Review | Narrative review | NA | NA |
| Paul et al, 2018 | SSA not specified | Belgium | Academic | Belgium | Academic | NA | Primary research | Qualitative | Interview study | global expert |
| Perumal-Pillay et al, 2017 | South Africa | South Africa | Academic | South Africa | Academic | pharmaceuticals | Primary research | Qualitative | Interview study | policy maker, academic |
| Poluta, 2006 | South Africa | South Africa | Academic | NA | NA | medical devices | Review | Narrative review | NA | NA |
| Rodriguez et al, 2017 | SSA: South Africa, Zambia | USA | Academic | USA | Academic | NA | Primary research | Qualitative | Interview study | policy maker, academic |
| Shillcutt et al, 2009 | LMIC not specified | USA | Academic | UK | government | NA | Review | Narrative review | NA | NA |
| Uneke et al, 2017 | Nigeria | Nigeria | Academic | Cameroon | Academic | NA | Review | Narrative review | NA | NA |
| Yong et al, 2015 | SSA: South Africa | USA | Consultant | USA | Consultant | NA | Review | Narrative review | NA | NA |
| Zegeye et al, 2017 | Ethiopia | South Africa | Academic | South Africa | Academic | NA | Primary research | Qualitative | Interview study | policy maker |
